# Oral benign fibrous histiocytoma: two case reports

**DOI:** 10.1186/1757-1626-2-9343

**Published:** 2009-12-17

**Authors:** Dardo Menditti, Luigi Laino, Antonio Mezzogiorno, Sara Sava, Alexander Bianchi, Giovanni Caruso, Luigi Di Maio, Alfonso Baldi

**Affiliations:** 1 Dipartimento di Scienze Odontostomatologiche, Seconda Università di Napoli, 80138 Napoli, Italia; 2 Dipartimento di Medicina Pubblica e Preventiva, Seconda Università di Napoli, 80138, Napoli, Italia; 3 Dipartimento di Biochimica, Sezione di Anatomia Patologica, Seconda Università di Napoli, 80138 Napoli, Italia; 4 Dipartimento di Chirurgia Toracica, Università di Chieti, 66100 Chieti, Italia

## Abstract

Fibrous histiocytoma is a benign soft tissue tumour arising as a fibrous mass everywhere in the human body. The involvement of the oral cavity is rare. We report two cases of benign fibrous histiocytoma that localized in the oral cavity. The clinical and histological features of the lesion are reported. Finally, a literature revision of this pathology at the level of the oral cavity is reported.

## Introduction

The term malignant fibrous histiocytoma (MFH) describes a soft tissue sarcoma arising from histiocytes and fibroblasts and many synonyms have been used with regard to potential malignancy [1-4]. The commonest sites affected by this tumour are: upper and lower limbs, orbit, retroperitoneum, pelvis, knee, head and neck. The development of immunohistochemical techniques and electronic microscopy during the past thirty years has allowed to discriminate between malignant and benign forms, consequently benign fibrous histiocytoma (BFH) became a clinical entity, although many synonyms are still used [1,5,6].

Nowadays, BFH is included in the so-called "fibrohistiocytic tumours of the soft tissues" that are divided into cutaneous and non-cutaneous types, and in the "fibrohistiocytic tumours of the bone" [4-6]. BFH is reported at any age with predominance in males adults (2.5:1) older than 25 years and with a mean age of 40 years [1-4]. The clinical features of the oral BFH are those of a painless solitary tumour, slowly enlarging, from 2-3 cm up to more than 10 cm, over a period of several months [1,4,6,7]. Symptoms include dysphagia, dyspnoea and, when the mass is located in the tongue, difficulty to speak may be present. The CT may be requested if it seems to be bone involvement. The treatment of choice to oral BFH is en-block surgical excision. The prognosis is good and the oral BFH recurs only if incompletely excised. Metastasis of the oral BFH have not been reported. However, it is recommended a regular period of clinical follow-up [1,4,6,8-12]. In the current paper it was reported the clinical and histological features of two cases of oral BFH.

## Case presentation

### Case report 1

**Figure 1:**
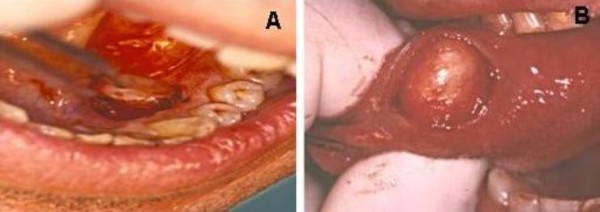
**(A) Macroscopical features of Case 1 at surgical excision**. **(B) **Macroscopical features of Case 2 at surgical excision.

**Figure 2:**
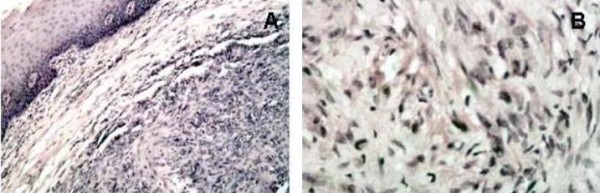
**(A) Histological appearance of the lesion in Case 1: the neoplasm is rich in cells of mesenchymal origin; stroma presents myxoid and hyalinization aspects and small foci of necrosis, while fibrous-histiocitic cells displays a storiform or cartwhell pattern (Haemotxylin and Eosin, original magnification ×20)**. **(B) **Higher magnification of figure 2A, better showing the cartwhell pattern of the fibrous-histiocitic tumour cells (Haematoxylin and Eosin, original magnification ×40).

**Figure 3:**
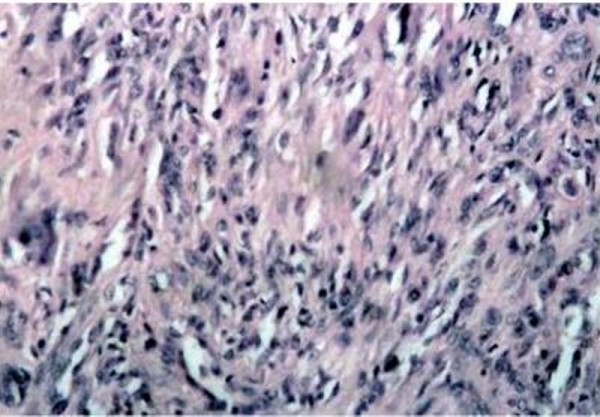
**Histological appearance of the lesion in Case 1: the neoplasm consists of a sub-mucosal, cellular aggregation of spindle-shaped, fibroblast-like cells with relatively pale, ovale nuclei; scattered round histiocytic cells are also present (Haemotxylin and Eosin original magnification ×20)**.

**Figure 4:**
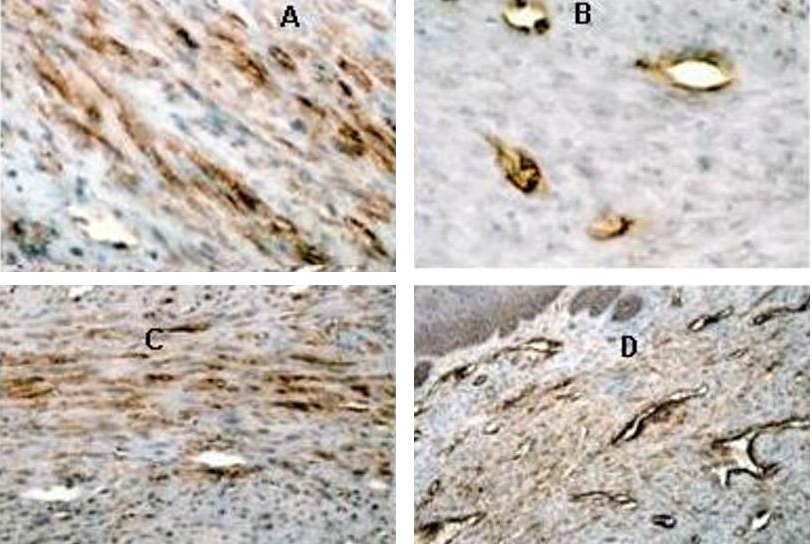
**(A) Strong immunohistochemical expression of CD68 in neoplastic cells in Case 1 (ABC, original magnification ×20)**. **(B) **Immunohistochemical expression of Factor XIIIa in Case 1: only vessels are positive (ABC, original magnification ×40). **(C) **Strong immunohistochemical expression of Vimentin in neoplastic cells in Case 2 (ABC, original magnification ×20). **(D) **Immunohistochemical expression of CD34 in Case 2: only vessels are positive (ABC, original magnification ×20)

In October 1998, a healthy 44-year-old male (Italian, white Caucasian) was referred to our department of oral surgery for evaluation and treatment of a tumour involving the lingual mucosa of the left mandible in the premolar area. Intra-oral examination disclosed a nodular and sessil mass 3.0 cm in diameter; the overlying mucosa appeared normoemich, normotrophic and not haemorrhaging. No lymph nodes were palpable. There were no other abnormalities in the oral cavity and the systemic conditions of the patients were good. On palpation the lesion was not painful and of fibro-elastic consistency. The clinical appearance of the lesion suggested the possibility of a neoplasm of soft tissues. The treatment of choice was radical excision of the tumour with 2-mm free margins (Figure 1). There was no relationship between the tumour and the underlying bone. The wound was primarily closed with 2-0 silk suture. Antibiotic coverage and chlorhexidine gluconate were prophylactically used. The post operative course was uneventful. The specimen consisted of an encapsulated mass measuring 3 × 2.5 cm. Macroscopically it showed a regular grey-yellow-white mass with dark areas of haemorrhage having fibroelastic consistency. Histopathology examination showed a neoplasm rich in cells that appeared of mesenchymal origin; stroma presented myxoid and hyalinization aspects and small foci of necrosis, while fibrous-histiocitic cells displayed a storiform or cartwhell pattern (Figure 2). Nuclear pleomorphism or hypercromasia were rarely detected. Mitotic activity was evaluated and demonstrated less than 5 per 10 high power fields (Figure 3). Cells tumour was highly positive for vimentin and CD68 (Figure 4A) and negative for S100, CD34, Factor XIIIa (Figure 4B) and SMA.

### Case report 2

In January 1999, a healthy 34-year-old male (Italian, white Caucasian) was referred to our department because of a slow-growing tumour in the mucosa located in the right side of the tongue (Figure 1). The mass grew over the last nine months. Intraoral examination revealed a distinct tongue asymmetry. On palpation the lesion, measuring approximately 2 cm in diameter, was not painful and seemed to be well encapsulated, mobile and of a firm-elastic consistency. The overlying mucosa appeared grossly normal. No lymph nodes were palpable. There were no other abnormalities in the oral cavity and the systemic conditions of the patients were good. The clinical appearance of the lesion suggested the possibility of a neoplasm of soft tissues. The lesion was excised under local anaesthesia and was easily removed; with blunt dissection of the mucosa from the underlying tissues. The wound was closed primarily with the placement of 2-0 silk suture. Antibiotic coverage and chlorhexidine gluconate were prophylactically used. The post operative course was uneventful. The specimen consisted of an encapsulated mass measuring 3,0 by 2,5 cm; the cut surface showed a non-tender elastic-hard mass, well circumscribed, non attached to the lower tissue, with normal mucosal surface, not ulcerated and without erosion of the contiguous bone. Histologically it was characterized by a submucosal, cellular aggregation of spindle-shaped, fibroblast-like cells with relatively pale, ovale nuclei; scattered round histiocytic cells were also present. Cells tumour was highly positive for vimentin (Figure 4C) and CD68 and negative for S100, factor XIIIa and CD34 (Figure 4D) and SMA.

In both cases treated, it was obtained clinical resolution and without side-effects or complications. Both patients are disease-free after 10-year follow-up.

## Discussion

The mandibular and lingual sites are rare localizations for BFH. Among the lesions we observed, the one in the tongue was the biggest and the more defined. The most difficult site for surgery was that on the mandibular mucosa, because of the presence of the lingual nerve. Radical excision with wide margins was the treatment of choice. The aetiology of oral BFH is obscure. Chronic irritation, continuous trauma and spontaneous development have been reported for those located within the oral cavity [1,4,13-16].

The clinical diagnosis of oral BFH should shell out by clinical features as slowly enlarging, well-circumscribed lesion, no aggressive behaviour with overlying intact mucosa; however, at clinical level, the differential diagnosis with other soft tissue neoplasms is not possible. Histological examination as rare mitosis, absence of cellular atypia and immunochemistry patterns as high positivity for vimentin, CD38, factor XIIIa [1,4,6]. The differential histological diagnosis includes the neurofibroma: this tumour is identified by positivity of S-100 protein [11]. Some leiomyosarcoma are diagnosed incidentally when presumpted BFH are removed. The negativity for SMA could differentiate this tumour from true BFH [1,5]. Another lesion that can be differentiated from the BFH is dermatofibroma, so-called atypical-BFH [12]. Atypical-BFH has similar response to the immunochemistry but the first arises in the subcutaneous tissue and the second one arises in the deep tissue. In the soft tissues of the oral cavity the principal lesion that requires a differential histological diagnosis from BFH is malignant fibrous hystiocitoma (MFH). The immunophenotypes of these tumours aren't sufficient to make a differential diagnosis. Histological pattern is important: the high pleomorphism of the cells, the high mitotic activity, more than 5 per 10 high power fields, and infiltration of the capsule and into the surrounding tissue are present in MFH [7]. In the cases presented, the neoplasms were clearly defined at clinical analysis and there were no signs of local invasion. Therefore, we decided to immediately perform surgical excision, postponing imaging analyses (TC-scan and MRI) to determine eventually secondary localizations of the tumour.

The prognosis of oral BFH is very good. Metastases haven't been reported. Local recurrence is present when the excision is incomplete. Indeed, for the BFH of the buccal mucosa it's necessary that the specimen has wide margins; the simple enucleation of the tumour from the surrounding tissue may facilitate local recurrences [1,4,6,10-12]. In conclusion, it was described two cases of oral BFH, successfully diagnosed and managed by surgical excision.

## Consent

Written informed consent was obtained from the patient for publication of these two cases and accompanying images. A copy of the written consent is available for review by the Editor-in-Chief of this journal.

## Competing interests

The authors declare that they have no competing interests.

## Authors' contributions

DM, LL and SS performed the surgical procedures and contributed to the analysis of the clinical data. AM and GC performed the histological examination of the lesion. AB performed the histological examination of the lesion and was a major contributor in writing the manuscript together with DM. All authors read and approved the final manuscript.
